# Functional Outcomes of Patients with Primary Brain Tumors Undergoing Inpatient Rehabilitation at a Tertiary Care Rehabilitation Facility in Saudi Arabia

**DOI:** 10.3390/ijerph20064679

**Published:** 2023-03-07

**Authors:** Sami Ullah, Ahmad Zaheer Qureshi, Farooq Azam Rathore, Waqas Sami, Imad Saeed Moukais, Fatimah Saif Alibrahim, Ibrahim Ali Asiri, Ayman Alsuhaibani

**Affiliations:** 1 Department of Physical Medicine and Rehabilitation, King Fahad Medical City, Riyadh 11525, Saudi Arabia; 2 Department of Physical Medicine and Rehabilitation, Qatar Rehabilitation Institute, Doha P.O. Box 3050, Qatar; 3 Department of Rehabilitation Medicine, PNS Shifa Hospital, Karachi 75530, Pakistan; 4 College of Nursing, QU Health, Qatar University, Doha P.O. Box 2713, Qatar; 5 Department of Orthopedics, King Saud University Medical City, Riyadh 12372, Saudi Arabia

**Keywords:** brain tumor, rehabilitation, length of stay, functional outcomes, Saudi Arabia

## Abstract

Rehabilitation services play a crucial role in improving the functionality and quality of life of individuals with a brain tumor; however, outcomes of inpatient rehabilitation based on tumor characteristics are not well known in the literature. This study was carried out to evaluate the effects of tumor characteristics on functional outcomes. A retrospective chart review was conducted for all adults with a diagnosis of primary brain tumor admitted for IPR between January 2014 and December 2019. Information was collected regarding demographics, characteristics of primary brain tumors, length of stay (LOS) and Functional Independence Measurement (FIM) scores. There were 46 patients, with the majority being male. The most common brain tumors were glioblastoma multiforme and meningioma. The mean LOS was 47.93 ± 26.40 days and the mean FIM gain was 78 ± 14. The type, grade and location of primary brain tumors did not show a significant correlation with the length of stay and functional gains during inpatient rehabilitation. There was a positive correlation between the FIM at admission and discharge, and a significant inverse correlation between the FIM score at admission and LOS. In-patient rehabilitation improved the functional outcomes in adult patients with primary brain tumors. Strategies to incorporate IPR in the care continuum of patients with brain tumors need to be adapted to improve regional services.

## 1. Introduction

With recent advances in cancer management, the survival rates of individuals with primary tumors of the brain have considerably improved in the last few decades [1,2]. The 5-year survival of patients with malignant brain tumor has been reported to be up to 38% [3]; however, with improved survival comes the importance of dealing effectively with long-term disabilities [4]. Cancer survivors can have several issues which adversely affect their mobility, community participation and quality of life (QOL). These include cognitive dysfunction, cancer-related fatigue, poor sleep, pain, neuropathy, lymphedema and radiation fibrosis syndrome [5,6,7]. Furthermore, factors related to medical complications of cancer affect the continuity of care [8]. This renders the need to institute a cancer rehabilitation program, which involves a comprehensive approach involving a team of rehabilitation experts working alongside the patients and families to facilitate the physical, psychological, social and vocational functioning of cancer survivors [6]. In individuals with brain tumors, disability or activity limitations are mainly secondary to neurological impairments caused by direct effects of brain tumors or related surgeries [8]. The most common neurological impairments in individuals with a brain tumor undergoing inpatient rehabilitation (IPR) are impaired cognition, weakness, visual–perceptual deficit, sensory loss and bowel and bladder dysfunction [9]. Other impairments include cranial nerve palsy, dysarthria, dysphagia, aphasia, ataxia and diplopia [10]. In addition to the deficits secondary to mass effects of tumors on the brain, there are other factors which can affect the functional outcomes such as age, disease onset, socio-economic status, accessibility to care and tumor characteristics (type, grade, location) [11]. In the literature, these factors are less often considered to be the direct determinants of functional prognosis in individuals with primary brain tumors. 

Saudi Arabia is the largest country in the Gulf region with an estimated population of around 34 million [12]. The prevalence of brain tumors in Saudi Arabia is the highest in the region after Iran [13], with the highest number of cases in the Riyadh province [14]. Brain tumors are considered to be the 10th most common cancer in adult males and females in the country and predominantly affect adults between 40 and 49 years of age [14,15]. Although primary brain tumors are relatively uncommon, representing only 1.6% of all cancers, they are associated with significant disability and mortality [1]. Non-malignant tumors were reported to be common in the adult population, while malignant tumors were more frequent in the pediatric population. In both groups, gliomas constituted the most common neoplastic category [16]. A study conducted in one of the tertiary hospitals in Saudi Arabia between 2015 and 2017 revealed that 5% of the patients had a moderate disability after surgery and 1.7% were in a vegetative or minimally responsive state [9]. Currently, dedicated cancer rehabilitation services are not available in Saudi Arabia. IPR services are located at only a few centers and the utilization of these services is suboptimal. There is a paucity of published research on the role of interdisciplinary inpatient cancer rehabilitation in patients with primary brain tumors in the country. Although there are articles highlighting the possible neurological outcomes of brain tumors depending on their type, grade and location, the effects of these tumor characteristics on LOS and functional outcomes during IPR are rarely reported [11,17,18,19]. 

The current study documents the effects of IPR on the functional outcomes in individuals with primary brain tumors at one of the largest tertiary care rehabilitation centers in Saudi Arabia. We also aimed to evaluate the effects of tumor characteristics, including type, grade and location of the brain tumor, on the LOS and functional outcomes during IPR. In addition, the histopathological diagnosis, tumor in relation to tentorium and anatomical site were included, which have rarely been documented in the literature on rehabilitation outcomes [20].

## 2. Materials and Methods

### 2.1. Setting 

A retrospective chart review was conducted for medical records of individuals with a primary brain tumor admitted for IPR at the Department of Physical Medicine and Rehabilitation, King Fahad Medical City (KFMC), Riyadh, Saudi Arabia between January 2014 and December 2019. The Rehabilitation Hospital at KFMC is the largest inpatient rehabilitation facility under the Ministry of Health which has dedicated comprehensive programs. The acquired brain injury unit is an 18-bed unit, which offers comprehensive integrated interdisciplinary inpatient and outpatient rehabilitation services to individuals with traumatic and non-traumatic brain injuries, including brain tumors. Although there are no dedicated beds for individuals with brain tumors, a considerable number of referrals are received from within the institute throughout Saudi Arabia for specialized neurosurgery and oncological treatment. Patients with brain tumors who can actively participate in 3 h of daily therapies and require at least 2 therapy services (out of physical therapy, occupational therapy and speech therapy) can be eligible for IPR program. An assessment of medical necessity and appropriateness of inpatient rehabilitation is determined by a rehabilitation physician through consultations. 

### 2.2. Inclusion Criteria

We included all adult patients (age > 18 years) of both genders with a confirmed diagnosis of primary brain tumor admitted to the inpatient rehabilitation facility at KFMC over a period of five years. Data regarding primary brain tumors and their anatomical locations were gathered from a retrospective chart review. Patients who fulfilled criteria for the grading system (Grade I–IV) for primary brain tumors as outlined by the World Health Organization (WHO) for central nervous system tumors [21] were included and assessed by a surgeon/oncologist.

### 2.3. Exclusion Criteria

Patients who died during hospital admission or were transferred back to the acute care were excluded.

The study was approved by the Institutional Review Board. At KFMC, electronic medical records of patients are maintained in a secure and dedicated database. The data were extracted using a pre-designed data extraction sheet. The data recorded included basic demographics (age, gender, marital status, occupation and mode of referral), characteristics of primary brain tumors (tumor name, type, grade, location, onset of disease, site, length of stay (LOS) and functional outcomes). The oncological treatment received until the time of admission to IPR was recorded. Functional outcomes were assessed using the Functional Independence Measurement scale (FIM). FIM is an 18-item, clinician-reported scale that assesses function in six areas including self-care, continence, mobility, transfers, communication and cognition [22]. Each item is rated from 1 (requiring total assistance) to 7 (completely independent) and scores can be measured at admission up to discharge from inpatient rehabilitation [23]. The difference between the admission and discharge scores constitutes the FIM change or FIM gain. Outcomes were assessed at the time of admission and discharge. 

### 2.4. Statistical Analysis

Data were computed and analyzed using SPSS 26.0 (IBM Corp, Armonk, NY, USA). Percentages and frequencies were calculated to present a summary of baseline study parameters and the mean ± SD were presented for quantitative variables such as age and LOS. The normality of the quantitative variables was checked through one-sample Kolmogorov–Smirnov tests. Categorical variables are expressed as frequencies and percentages. One-Way Analysis of variance (ANOVA) was applied to observe mean differences among various categorical variables. A *p*-value of < 0.05 was considered statistically significant.

## 3. Results

We identified 46 patients admitted during the study period fulfilling the inclusion criteria. There were 27 (58.7%) males and 19 (41.3%) females. The mean age of the patients was 42.9 ± 18 years. Almost one quarter were employed fulltime, while 14 were unemployed. For most of the patients (40; 87%) it was their first admission to the inpatient rehabilitation center and the majority of patients (39; 84.9%) were directly transferred from within KFMC. The basic socio-demographics are presented in Table 1.

The tumor was located in the supratentorial region in 30 patients (65.2%) and the infratentorial region in 16 patients. Around half of the study participants had an onset of disease at less than one year, followed by more than three years for 11 patients, 1–2 years for 7 patients (15.2%) and 2–3 years for 5 patients. The majority of patients had frontal lobe tumors (*n* = 13; 28.3%), around 20% at the supratentorial meninges, 13% at the infratentorial meninges and 9% (*n* = 4) at the cerebellum. More than 50% of the patients had non-gliomas, whereas 43.5% (*n* = 20) had gliomas. Nearly 75% of the patients had low-grade tumors. According to the WHO classification, most patients had a grade 1 tumor (*n* = 20; 43.5%). Meningioma was the most common tumor type present in almost one quarter of patients, followed by glioblastoma multiforme (*n* = 11; 23.9%) and central neurocytoma (*n* = 5; 10.9%). More than 50% of the patients underwent tumor resection, followed by the combination of tumor resection and radiotherapy (*n* = 13; 28.3%) and approximately 20% were treated with a combination of tumor resection, chemotherapy and radiotherapy. The results are presented in Table 2.

The mean LOS was 47.93 ± 26.40 days while the FIM gain was 78 ± 14. No significant difference was observed between the LOS and educational status (*p* = 0.37), admission source (*p* = 0.15), onset of disease (*p* = 0.46), tumor location (*p* = 0.34), tumor category (*p* = 0.75), WHO grade (*p* = 0.74) and primary treatment (*p* = 0.60). Moreover, no significant correlation was observed between the LOS and FIM gain (r = −0.047, *p* = 0.75). In addition, no significant difference was observed between the FIM gain and education status (*p* = 0.26), admission source (*p* = 0.21), onset of disease (*p* = 0.66), tumor location (*p* = 0.67), tumor category (*p* = 0.55), WHO grade (*p* = 0.99) and primary treatment (*p* = 0.24) (Table 3). 

**Figure 1:**
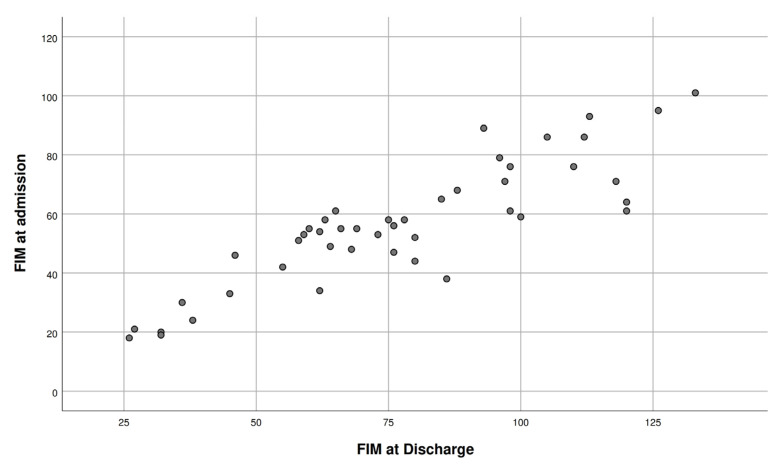
Positive correlation between FIM at admission and FIM at discharge.

**Figure 2:**
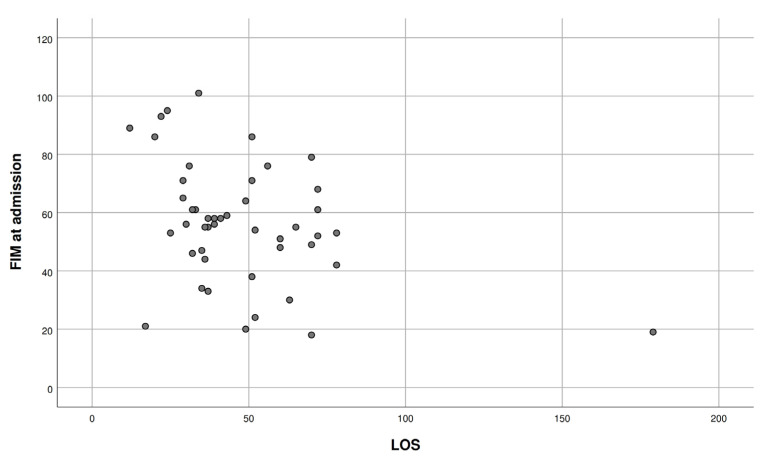
Inverse correlation between FIM at admission and LOS. FIM: Functional Independence Measure. LOS: Length of Stay.

No significant correlation was seen between the FIM gain and age (r = 0.276, *p* = 0.06); however, there was a positive correlation between FIM at admission and at discharge. (Figure 1). We also observed a significant inverse correlation between the FIM score at admission and the LOS (r = −0.387, *p* = 0.008) (Figure 2).

## 4. Discussion

As brain tumors progress, they result in significant disability and mortality [24]. Patients with brain tumors can develop a number of problems and complications leading to impairments. These could be attributed to the tumor itself or the adverse effects of treatment (surgery, radiotherapy and chemotherapy) [25]. Due to recent developments in diagnostics and treatment modalities, the survival of patients with a primary brain tumor has increased considerably over the past years [4]. The 5-year survival of patients with primary non-malignant brain tumors has been reported to be as high as 96% [3]; however, outcomes cannot be solely attributed to the oncological treatments such as surgery, chemotherapy and radiation therapy [26]. Factors beyond the disease-specific care need to be considered equally important. The role of rehabilitation throughout the course of treatment needs to be highlighted, which can vary from one health system to another. Conventionally, data regarding cancer management revolve around mortality, morbidity and survival analysis. Similarly, it is not uncommon to observe that when there is a new diagnosis of a serious condition such as a brain tumor, the focus remains on the diagnostics and treatment of disease process. Consequently, the aspects regarding rehabilitation of hospitalized patients as a part of the treatment plan remain underemphasized. This is important, as IPR is one of the important avenues for improving the function, quality of life and social empowerment of individuals with disability. Through this study, we intended to highlight that the word “prognosis” in brain tumors needs to be perceived beyond the definitions of mortality and survival. We believe that this general interpretation undermines the role of rehabilitation, which has an important role in the functional prognosis and quality of life in cancer survivors. This study adds to the growing literature on the role of interdisciplinary rehabilitation in patients with primary brain tumors. 

Rehabilitation during acute treatment of brain tumor emphasizes the prevention of deconditioning and secondary complications such as venous thromboembolism, skin ulcers, contractures, constipation and pneumonia [27]. It also encourages early mobilization and training to achieve a resumption of dependence in activities of daily living [28]. In cancer survivors, interdisciplinary rehabilitation has been shown to improve fatigue, communication and cognition [11,29]. The effects of cancer are not only limited to patients, as the caregiver burden is also an important aspect of cancer care. One study followed up caregivers of brain tumor patients for one to four years after their patients completed the rehabilitation program [30]. Caregivers were generally satisfied with the outcomes, particularly the motor, cognitive and activities of daily living skills. Since the care of individuals with brain tumor is multidirectional, experts have highlighted that brain tumor rehabilitation is a complex and challenging task [31]. In addition, there is a lack of high-quality research on the effectiveness of brain tumor rehabilitation in the form of well-designed randomized clinical trials and the methodology of available research is considered weak [32]. Research has shown that the functional deficits in patients with brain tumors attending IPR interventions are similar to patients with other neurological conditions such as stroke and traumatic brain injury [33,34,35]. Extrapolation of this concept supports a possible role for rehabilitation in patients with brain tumors [36]. 

The majority of patients in our study were male, married and employed. These findings may be of less significance in western health systems; however, the majority of the labor force in Saudi Arabia is male, which is also attributed to the cultural and social norms of the country [37]. More than a quarter of patients in this study had a frontal lobe tumor. The frontal lobe is involved in a wide range of “higher” cognitive functions including language, thinking, future planning, self-management and decision-making. Damage to the frontal cortex can adversely affect the social and community functioning of an individual due to difficulties with planning, executive functioning, attention, loss of memory and changes in personality [38]. A cancer rehabilitation program with integrated psychosocial services and cognitive rehabilitation remains crucial in brain tumor rehabilitation. 

Brain tumor rehabilitation can be delivered in both the inpatient [39] and outpatient settings [40]. It involves various disciplines which can be provided in a variety of settings and tailored to the needs of the individuals with a brain tumor. Rehabilitation by design is an active process that engages the patient and provides tools for the long-term management of disease-related impairments. When function cannot be completely restored, rehabilitation facilitates recovery and provides compensations for functional loss or adaption to the environment to optimize physical performance and participation in social roles [41]. This is particularly relevant for patients with a brain tumor who can benefit from and improve with these rehabilitation interventions. A major global challenge for brain tumor rehabilitation is the lack of cancer rehabilitation services, mainly in the developing world [42]. Where available, the underutilization of these services is also another factor [8,28]. A study on a large cohort of individuals with brain tumor patients from Italy showed that a limited number of patients received rehabilitation interventions during the first year after diagnosis [39]. Langbecker and Yates reported that many brain tumor survivors had unmet needs and were not aware of services available [43]. Only half of the 40 patients with primary brain tumors in their study were referred to rehabilitation services. Most of the referrals were limited to physiotherapists and speech therapists. The comparatively low referral rate to psychosocial services may limit patients’ abilities to cope with their condition and the changes they experience. Worku et al. identified the unavailability of supplies, lack of professionals and cost of service as the main barriers to receiving cancer rehabilitation services in Ethopia [42]. In addition, many oncologists and surgeons are unaware of the value of early coordinated inter-disciplinary rehabilitation for patients with brain tumors and do not make the necessary referrals [8]. This was also reflected in the current study, where the majority of the referrals for rehabilitation were from within our institute. There are limited rehabilitation venues for comprehensive rehabilitation in regions other than the Riyadh province. Since there is a considerable demand for rehabilitation care in the country for individuals with trauma, stroke and birth-related disabilities [44], the attention to dedicated brain tumor rehabilitation remains underemphasized. This may also be attributed to a lack of awareness of primary treating teams (oncologists/surgeons) about the scope of rehabilitation for cancer survivors in general [10,12,13]. An Italian study showed that only 12.8% of patients with brain tumors received inpatient rehabilitation and only 14.9% received outpatient rehabilitation services [39]. Only a few (13%) patients were admitted with recurrent brain tumors. In a comparative study between newly diagnosed glioblastoma and recurrent brain tumors, there was no statistically significant difference between the groups for FIM scores at admission or discharge; however, this was not analyzed in our study [45].

Primary brain tumors are generally classified based on their tissue of origin, with gliomas representing 40–60% of brain tumors [46]. This is similar to the findings in our study, which showed that 43.5% of patients had gliomas; however, subgroup analysis showed that of all brain tumors, meningioma (32.6%) and glioblastoma multiforme (23.9%) were the most common (Table 2). Data collected from U.S. cancer statistics (2012–2016) showed that meningioma constituted 37.6% of brain tumors, followed by sellar region tumors (17.5%) and glioblastoma (14.6%) [47]. Glioblastoma has been well studied given its aggressive nature and poor prognosis [11,47]. Meningiomas are generally less aggressive compared to other tumors, but given that they represent the vast majority of brain tumors, they pose a challenge due to the burden of care involved [48]. Although meningiomas are commonly benign, it is important to note that meningiomas can be malignant as well, as in our study one of the patients had Grade III meningioma [21]. Tumor characteristics in relation to functional outcomes during IPR have not been well studied and there are very few studies published in the literature. Roberts et al. reported that newly diagnosed patients with glioblastoma who undergo inpatient rehabilitation demonstrated significant functional improvements, primarily in the mobility domain [17]. After adjusting for confounding variables, there was no significant difference in survival times between the patients who went to inpatient rehabilitation and those who did not. Fu et al. reported that high-grade astrocytoma patients had longer lengths of stay and greater overall FIM gains than low-grade astrocytoma patients [49]. An average gain of 10 on the motor FIM score during rehabilitation was reported as a significant factor of longer survival in glioblastoma [50]. There were no statistically significant differences found in FIM efficiency in studies comparing low-grade with high-grade astrocytomas [49], nor when comparing astrocytoma, meningioma and metastatic brain cancer patients [18]. Although we did not use FIM efficiency as an outcome measure, our results showed that FIM gains were not significantly associated with tumor type, location and grade of tumors. This finding suggests that the scope of inpatient rehabilitation programs may not be limited to a particular type, grade or anatomical site of brain tumor; however, this recommendation cannot be generalized and the scope of practice needs to be individualized based on institutional resources. 

The average length of stay (LOS) in this study was 47.93 ± 26.4 days, which was comparable with the LOS previously reported in the literature (11.5 to 76.9 days) [8,40,51]. High-grade tumor patients tend to have a longer LOS [49]. Our study did not show a significant correlation between the LOS or FIM gain with different parameters including age, gender, educational status, tumor grade, location and mode of admission. Previous studies have suggested that the etiology of the brain tumor is not a significant factor in determining the improvements in the functional outcomes [9,33]. Other studies have reported improved functional outcomes both after inpatient or outpatient interdisciplinary rehabilitation. Fu et al., in their retrospective chart review of thirty patients with leptomeningeal disease, reported that patients who completed acute IPR made statistically significant improvements on the majority of FIM items [49]. Shahpar et al. enrolled 49 patients in a prospective longitudinal study reported improved functional outcomes with interdisciplinary outpatient rehabilitation program in individuals with malignant brain tumors [40]. The FIM score at the time of discharge in our study was higher than the score at admission. This supports the role for IPR; however, head-to-head studies comparing functional outcomes of IPR vs. outpatient rehabilitation are lacking. For patients undergoing IPR in our study, half of them had a disease onset of less than 1 year; however, given the finding that the same number of patients did not receive inpatient rehabilitation within one year of onset raises a concern. It needs to be further explored whether they did not require IPR within a year of onset or they did not get an opportunity for IPR. Patients who underwent IPR after 3 years of tumor onset constituted nearly a quarter of the patients. A key finding in our study is was the inverse correlation of FIM at admission and LOS. Patients with a higher FIM at admission had a lower LOS. The LOS is generally considered to be one of the most important performance indicators of hospital efficiency and cost of care. It is important for IPR, since the rehabilitation LOS is usually longer than the stay in acute care. This finding emphasized the role of early rehabilitation during acute care prior to admission to IPR, including rehabilitation during intensive care, peri-operative care or rehabilitation during initial chemotherapy and radiotherapy. This will condition the patient to a higher level of function at admission to IPR, which may contribute towards higher functional gains within a shorter LOS, as supported by our findings. 

There are some strengths and weaknesses of the study which warrant mention. This study provided unique insights into the inpatient rehabilitation services, detailed demographics with in-depth classification of patients with primary brain tumors and referral patterns in Saudi Arabia. There is a paucity of literature on the role of acute and subacute inpatient or outpatient multidisciplinary rehabilitation in patients with primary brain tumors. Most of the local studies have used survival and tumor recurrence as outcome measures. There is a need to conduct well-designed studies to evaluate the functional outcomes of adult patients with primary brain tumors undergoing both an inpatient and outpatient rehabilitation program. There is also a need to evaluate if community rehabilitation can help maintain the functional gains achieved during IPR. Such studies will help evaluate the setting, intensity and duration of the existing rehabilitation protocol and, as a result, identify the best local practices and guide efficient planning and resource allocation. 

### Limitations 

We were unable to document the initial presentation in these patients, which can vary according to the site and grade, and may influence the rehabilitation outcomes. Surgical details were also not included, which could have potentially affected functional outcomes. The lack of access to medical records for patients operated on at other institutes was a limitation in this regard. A future study with prospective data collection might yield different results. The location of the brain tumor was recorded based on the gross anatomy of the brain; however, their precise location based on functional neuroanatomy could have been more useful in order to find correlations with functional outcomes. Moreover, FIM subcategories were not included as outcomes in this study. Although our study showed that the type, grade and location of primary brain tumors did not show significant correlations with the length of stay and functional gains during inpatient rehabilitation, due to the small sample size and predominance of low-grade tumors in our study population, this statistical significance cannot be considered reliably conclusive and should be interpreted with caution. 

## 5. Conclusions

In-patient rehabilitation improves the functional outcomes in patients with a primary brain tumor. Multicentered studies with a larger cohort of patients are required to determine the effects of brain tumor characteristics on outcomes of IPR. Since functional status at admission to IPR affects the LOS, there is a need to focus on acute care rehabilitation. This study has brought attention to improving cancer rehabilitation services and its awareness among health care providers and brain tumor survivors. Strategies to incorporate IPR in the care continuum of patients with brain tumors need to be adapted to improve regional services.

## Figures and Tables

**Table 1:** Socio-demographic characteristics of the patients (*n* = 46).

Variables	*N* (%)	Variables	*N* (%)
**Education Level** College Illiterate Intermediate School Primary School Secondary School	17 (37.0) 11 (23.9) 6 (13.0) 4 (8.7) 8 (17.4)	**Admission to Rehabilitation** First Second Third	40 (87.0) 4 (8.7) 2 (4.3)
		**Admission Source** Direct transfer from another hospital Direct transfer from within KFMC Elective admission from home	2 (4.3) 39 (84.8) 5 (10.9)
**Occupation** Age Retirement Disability Retirement FT Employed Student Unemployed	6 (13.0) 2 (4.3) 16 (34.8) 8 (17.4) 14 (30.4)		
		**Marital Status** Divorced/Separated Married Single Widow	1 (2.2) 27 (58.7) 15 (32.6) 3 (6.5)

**Table 2:** Tumor characteristics and distribution of gliomas and non-gliomas among WHO tumor grades and tumor type.

Category	Total *n* (%)	Tumor Type	WHO Tumor Grade *n* (%)		Brain Tumor *n* (%)
**Tumor Type** Gliomas Non-Glioma	20 (43.5) 26 (56.5)	Glioma 20 (43.5%)	Low Grade 9 (45%)	Grade I 4 (44.4%)	Pilocytic astrocytoma (4; 44.4%)
**High/Low Grade** High Grade Low Grade	12 (26.1) 34 (73.9)			Grade II 5 (55.6%)	Diffuse Astrocytoma (2; 40%) Pure oligodendroglioma (1; 20%) Oligoastrocytoma (1; 20%) Ganglioglioma (1; 20%)
**WHO Grade** Grade 1 Grade II Grade III Grade IV	20 (43.5) 14 (30.4) 1 (2.2) 11 (23.9)		High Grade 11 (55%)	Grade III 0 (0.0)	None
**Tumor ** “Diffuse” Astrocytoma Cavernous Hemangioma Central neurocytoma Craniopharyngioma Ganglioglioma Gliobastoma multiforme Menignioma Oligoastrocytoma Pilocytic astrocytoma Pure oligodendroglioma Schawannoma	2 (4.3) 3 (6.5) 5 (10.9) 1 (2.2) 1 (2.2) 11 (23.9) 15 (32.6) 1 (2.2) 4 (8.7) 1 (2.2) 2 (4.3)			Grade IV 11 (100%)	Gliobastoma multiforme (GBM) (11; 100%)
**Primary Treatment** Tumor resection Tumor resection, chemotherapy and radiotherapy Tumor resection and radiotherapy	24 (52.2) 9 (19.6) 13 (28.3)	Non-Glioma 26 (56.5%)	Low Grade 25 (96.2%)	Grade I 16 (64%)	Craniopharyngioma (1; 6.3%) Menignioma Grade I (10; 62.5%) Schawannoma (2; 12.5%) Cavernous Hemangioma (3; 18.8%)
**Tumor Location** Infratentorial Supratentorial	16 (34.8) 30 (65.2)			Grade II 9 (36%)	Meningioma Grade II (4; 44.4%) Central Neurocytoma (5; 55.6%)
**Onset of Disease** <1 year 1–2 years 2–3 years >3 years	23 (50.0) 7 (15.2) 5 (10.9) 11 (23.9)		High Grade 1 (3.8%)	Grade III 1 (100%)	Meningioma Grade III (1; 100%)
**Tumor Site** Cerebellum Frontal lobe Infratentorial Meninges Medulla Mid Brain Parietal lobe Pons Sellar region Supratentorial Meninges Temporal lobe Thalamus	4 (8.7) 13 (28.3) 6 (13.0) 2 (4.3) 2 (4.3) 2(4.3) 2 (4.3) 1 (2.2) 9 (19.6) 3 (6.5) 2 (4.3)			Grade IV 0 (0.0)	None

WHO: World Health Organization.

**Table 3:** Comparison of LOS and FIM gain with different study parameters.

Parameters	LOS (Days)	*p*-Value	FIM Gain	*p*-Value
**Education Status** Illiterate Primary Secondary Intermediate College	37.09 ± 7.06 42.25 ± 19.01 54.38 ± 23.40 38.67 ± 18.93 55.56 ± 36.89	0.378	26.91 ± 16.96 16.25 ± 8.26 11.63 ± 6.14 18.67 ± 16.40 23.13 ± 13.88	0.263
**Admission Source** Direct transfer from another hospital Direct transfer from within KFMC Elective admission from home	28.00 ± 15.55 51.13 ± 27.27 31.00 ± 7.960	0.153	11.50 ± 7.77 22.23 ± 14.49 12.40 ± 14.03	0.212
**Onset of Disease** <1 year 1–2 years 2–3 years >3 years	47.35 ± 18.43 35.14 ± 12.42 54.40 ± 12.38 54.36 ± 45.33	0.469	19.57 ± 15.12 27.00 ± 11.13 19.40 ± 11.12 20.00 ± 15.06	0.667
**Tumor Location** Infratentorial Supratentorial	53.00 ± 38.82 45.23 ± 16.76	0.348	19.56 ± 14.56 21.43 ± 13.95	0.672
**Tumor Category** Gliomas Non-Glioma	46.55 ± 18.65 49.00 ± 31.43	0.759	19.80 ± 14.98 21.54 ± 13.51	0.682
**W.H.O Grade** Grade 1 Grade II Grade III Grade IV	45.85 ± 35.27 51.21 ± 17.32 72.00 ± 0.00 45.36 ± 17.16	0.748	20.55 ± 13.51 21.36 ± 14.63 20.00 ± 0.00 20.55 ± 16.13	0.998
**Primary Treatment** Tumor resection Tumor resection, chemotherapy and radiotherapy Tumor resection and radiotherapy	45.42 ± 32.86 45.44 ± 15.92 54.31 ± 17.73	0.600	17.46 ± 10.89 25.22 ± 17.02 23.85 ± 16.45	0.243

LOS: Length of stay. FIM: Functional Independence Measure.

## Data Availability

The data presented in the study are available upon request from the corresponding author.
